# Predicting nanomaterials pulmonary toxicity in animals by cell culture models: Achievements and perspectives

**DOI:** 10.1002/wnan.1794

**Published:** 2022-11-23

**Authors:** Emilio Di Ianni, Nicklas Raun Jacobsen, Ulla Vogel, Peter Møller

**Affiliations:** ^1^ National Research Centre for the Working Environment Copenhagen Denmark; ^2^ National Food Institute Technical University of Denmark Kongens Lyngby Denmark; ^3^ Department of Public Health, Section of Environmental Health University of Copenhagen Copenhagen Denmark

**Keywords:** advanced cell culture models, DNA damage, inflammation, nanomaterials, predictivity

## Abstract

Animal experiments are highly relevant models for the assessment of toxicological effects of engineered nanomaterials (ENMs), due to lack of biomonitoring and epidemiological studies. However, the expanding number of ENMs with different physico‐chemical properties strains this approach, as there are ethical concerns and economical challenges with the use of animals in toxicology. There is an urgent need for cell culture models that predict the level of toxicological responses in vivo, consequently reducing or replacing the use of animals in nanotoxicology. However, there is still a limited number of studies on in vitro–in vivo correlation of toxicological responses following ENMs exposure. In this review, we collected studies that have compared in vitro and in vivo toxic effects caused by ENMs. We discuss the influence of cell culture models and exposure systems on the predictability of in vitro models to equivalent toxic effects in animal lungs after pulmonary exposure to ENMs. In addition, we discuss approaches to qualitatively or quantitatively compare the effects in vitro and in vivo. The magnitude of toxicological responses in cells that are exposed in submerged condition is not systematically different from the response in cells exposed in air–liquid interface systems, and there appears to be similar ENMs hazard ranking between the two exposure systems. Overall, we show that simple in vitro models with cells exposed to ENMs in submerged condition can be used to predict toxic effects in vivo, and identify future strategies to improve the associations between in vitro and in vivo ENMs‐induced pulmonary toxicity.

This article is categorized under:

Toxicology and Regulatory Issues in Nanomedicine > Toxicology of Nanomaterials

## Graphical abstract

This review describes current evidence of the predictability of cellular models to equivalent toxicological responses in animals following pulmonary exposure to nanomaterials, and outlines perspectives on refinement of in vitro approaches to predict hazardous properties of inhaled nanomaterials to human health.

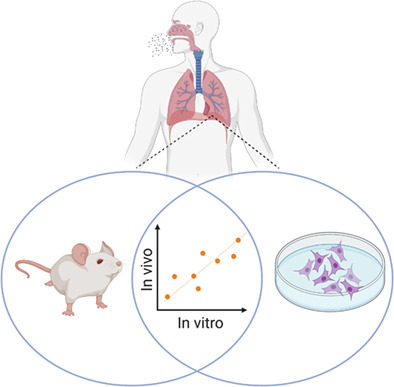

## 1 INTRODUCTION

Engineered nanomaterials (ENMs) are increasingly used for industrial, consumer, and medical applications, resulting in increased human exposure. Greatest exposure occurs in the working environment, with inhalation being the main route of exposure. Because of their small size, inhaled ENMs deposit in the distal part of the respiratory tree regions of the lung and generate inflammation, oxidative stress, and damage to biomolecules such as lipids and DNA (Møller et al., 2014). In turn, these effects activate a cascade of events leading to diseases such as fibrosis, lung cancer, and arteriosclerosis (Hadrup et al., 2020; Halappanavar et al., 2020; Stone et al., 2017). Diseases such as cancer and atherosclerosis may develop after long‐term exposure by either repeated exposure to materials with “high” clearance rate (e.g., carbon black) or even single exposure to materials with “low” clearance rate (e.g., carbon nanotubes). The dose determines the debut time and severity of the disease. In general, in vitro models cannot yet recreate the formation of a tumor or atheroma because these clinical findings develop through multiple steps. In addition, there is as yet no single intermediate step that has proven to be necessary or a sufficient causal factor for ENM‐induced diseases. In vitro models are based on the current knowledge on the mechanism of action for ENM‐induced health effects.

Safety assessment of ENMs is an essential element in safe‐by‐design approaches, which introduces toxicological testing in early steps of research and development to prevent adverse health effects in exposed workers. Animal experiments is the traditional way of studying toxicological effects of ENMs since evidence from epidemiological studies is not (yet) available for these new materials. Furthermore, available epidemiological studies of occupational exposure to high volume production nanomaterials such as carbon black and titanium dioxide suffer from limitations such as lack of information on particle size and thus, the magnitude of nanoparticle exposure (Saber et al., 2019). A typical experiment performed according to Organization for Economic Co‐operation and Development (OECD) guidelines for testing of chemicals entails multiple doses and time‐points, each group having up to 10 animals, resulting ultimately in large numbers of animals used to assess a substance (e.g., 120 animals per ENM). Study designs can be developed to reduce the number of animals, but it still sums up to a rather large number of animals, thus colliding with the principles of Replacement, Reduction, and Refinement (3Rs) of animal testing, which have been developed to perform more humane animal research. Alternatives to animal testing is an ongoing theme in nanotoxicology. There are huge benefits of developing robust in vitro models for screening toxicological effects of ENMs in cell cultures before embarking on more advanced and expensive animal models. These benefits can be summarized as follows:

1. Animal testing may only be needed if ENMs induce toxic effects in cell cultures (i.e., screening of hazardous ENMs).
2. Cell culture models can be used to estimate the required group size in animal studies.
3. Cell culture studies can be used to select the dose range in animal studies.
4. For non‐toxic ENMs in cell cultures, it may not be necessary to perform a full dose–response relationship (i.e., a no observed adverse effect level can be established using information about realistic human exposures and fewer doses and time‐points in an animal study).

Despite the large number of studies assessing the toxicity of ENMs in cell cultures, there are few studies that have investigated whether equivalent responses are obtained in cell cultures and animals after exposure to the same ENMs. Regulatory authorities still rely on in vivo studies of ENM toxicity to establish exposure levels with tolerable risk of health effects for workers. There is a need for reliable in vitro testing systems that predict in vivo responses following ENM exposure.

Studies on the relationship between toxicological effects in cell cultures and animals may follow different paths, depending on the assessed research question. The simplest question is whether the responses in vitro and in vivo are the same (i.e., concordance based on binary categorization of responses). However, this question is typically complicated by the fact that it is the dose of ENMs that mainly determines the level of effect. Thus, very high doses may produce a statistically significant effect, but may have little biological relevance. It introduces a somewhat arbitrary factor of selecting an upper boundary of doses, which may or may not be regarded as realistic doses. A more elegant approach is to compare the dose–response relationship in cell culture and animal studies, although this entails some assumptions regarding the shape of the dose–response curves. This analysis uses either categorical or continuous data from cell culture models to predict the response in animals and ultimately in humans. Another important issue is the type of cell culture model. The traditional in vitro experiment entails a monoculture of immortalized or primary cells. Cell lines are robust, easy to culture, and are relatively cheap. Thus, they are broadly used, which increases possibilities of interstudy comparison of toxicological effects of ENMs. However, immortalized cells generally have aberrant karyotype and increased genomic instability as compared with the tissue cells from which they originated. On the contrary, early passages of primary cells have the same genotypic and phenotypic characteristics as tissue cells and better represent the healthy microenvironment. Yet primary cells are typically costly to produce, have low proliferation rate, less robust, and usually more laborious to work with.

**FIGURE 1:**
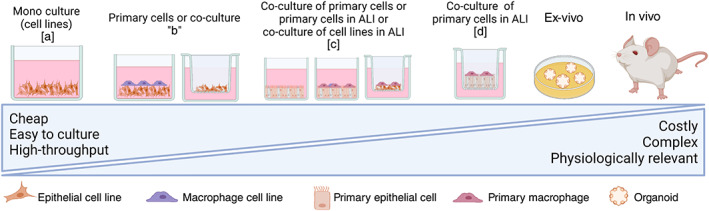
Pulmonary cell models of increasing complexity. Monocultures of cell lines [a] are cheaper, easier to culture, and have higher throughput valuable for battery tests of multiple compounds and endpoints. Increasing the complexity to [b] through [d], and up to ex vivo, the physiology of the model becomes higher and closer to that of an in vivo animal model. Increasing complexity increases the costs too

Advanced cell culture models are explored in particle toxicology as more physiologically relevant exposure systems as compared with monocultures of cells exposed to ENMs in submerged condition. These include co‐cultures (i.e., more than one cell type), 3D cultures (systems where cells are allowed to form a three‐dimensional structure rather than a monolayer) and special exposure systems such as air–liquid interface (ALI). The latter includes particle exposure in vertical (i.e., sedimentation of particles from a cloud) and horizontal direction (i.e., sedimentation of particles from radial flow of air over the cellular layer). There are two commercially developed ALI systems—called INVITROCELL and CULTEX—as well as a number of non‐commercial exposure systems (Upadhyay & Palmberg, 2018). Further description of difference between cell culture models has been elaborated previously (Evans et al., 2017). It is still unclear whether these advanced cell culture models are superior to the traditional monoculture systems. In this regard, cost–benefit considerations should be kept in mind as simple monoculture systems are relatively cheap and the added value of more complex systems needs to be carefully assessed. Figure 1 shows the differences between traditional monocultures, co‐cultures, 3D cultures and ALI. The increasing complexity of the cell culture systems are categorized into four groups corresponding to the physiological relevance relative to the exposure condition in human airway epithelial cells.

The scope of this review is to discuss advances in the field of in vitro nanotoxicology, with emphasis on exposure systems and cell models to mimic the lung cell architecture, yet some aspects concerning the in vitro–in vivo extrapolation are applicable to other physiological compartments such as gastrointestinal tract, brain, and skin. There is not a sufficient number of studies on toxicity caused by ENMs in humans, therefore hazard identification relies currently on in vitro and in vivo studies. The review encompasses three sections that address key questions in research on in vitro–in vivo comparisons of health hazard identification by exposure to EMNs:

1. Can cell culture models predict pulmonary toxicity in animals?
2. Does the magnitude of response in cell cultures correspond to the magnitude of pulmonary toxicity in animals?
3. Are advanced cell culture models better predictors of pulmonary toxicity in animals as compared with mono‐cultures?

## 2 METHODS

We have searched the PubMed database for studies on ENMs that have compared in vitro with in vivo endpoints upon exposure to ENMs. Inclusion criteria of studies have been: (1) ENMs have been characterized with regard to particle size, surface area, and so on; 2) at least three ENMs have been assessed in cells and animals; (3) comparison of the in vitro–in vivo relationship was a primary objective of the study. Eighteen studies have been included in the review (listed chronologically in Table 1). The comparisons are based on metal oxides and SiO_2_ (13 studies), and carbonaceous nanomaterials including elemental carbon, carbon black, multiwalled carbon nanotubes, and graphene oxide (9 studies). The endpoints encompass biomarkers of cytotoxicity, oxidative stress, inflammation, and DNA damage. In the review, we distinguish between “endpoint” (i.e., specific biomarker) and “response” (i.e., inflammation). The same response can be assessed using different endpoints in cells and animals.

**TABLE 1:** Summary of studies comparing qualitatively or quantitatively the pulmonary toxicity between in vitro and in vivo models

Authors	Nanoparticles	Cell models and exposure system endpoints	Animal models and exposure endpoints	Outcome
Sayes et al. (2007)	Carbonyl iron (CI, 800–3000 nm), crystalline silica (CS, 1600 nm), precipitated amorphous silica (AS, 1000–3000 nm), nano‐ZnO (50–70 nm), fine‐ZnO (<1000 nm)	Rat lung epithelial cells (L2) primary alveolar macrophages (AMs) L2‐AMs co‐culture Submerged (4, 24, and 48 h) Inflammation (MIP‐2, TNF‐α, and IL‐6), cytotoxicity (LDH, MTT)	Fisher 344 rats Intratracheal Instillation (24 h, 1 week, and 3 months) Neutrophil influx and LDH in BALF lung histology	No correlation between in vitro and in vivo endpoints across the time points The method developed later by Rushton et al. (2010) was applied to data generated in this study. This showed strong correlations between LDH or MIP‐2 in AMs or L2‐AMs co‐culture, respectively, and neutrophil influx after doses were normalized by surface area
Warheit et al. (2009)	ZnO (nanosize; 50–70 nm; fine size: <1000 nm)	Lung epithelial cell line (L2), primary alveolar macrophages (AMs), or L2‐AMs co‐culture Submerged (4 and 24 h) LDH, MIP‐2, and TNF‐α	Male Crl:CD(SD)IGS BR rats Inhalation and intratracheal instillation (24 h, 3 days, and 8 days) Neutrophil influx and LDH in BALF	No correlation between in vitro and in vivo proinflammatory responses
Rushton et al. (2010)	Elemental carbon (41 nm); TiO_2_ (20‐250 nm); Cu (40 nm); Ag (35 nm); Au (50 nm); polystyrene‐NH_3_ (60 nm)	A549 Luc1cells (stably transfected with an IL8‐luciferate reporter construct) alveolar macrophages Submerged Electron spin resonance (ESR), IL8	Male Fisher 344 rats Intratracheal instillation (24 h) Neutrophil influx in BALF	In vivo PMN response was strongly correlated with AM ESR (DMPO spin trapping) activity (*r*_*s*_ = 0.97, *p* < 0.001) and A549 Luc‐1 cell luciferase response (*r*_*s*_ = 0.94, *p* < 0.01)
Wang et al. (2011)	Pristine and functionalized (COOH) MWCNTs (length: 10–30 μm; diameter: 20–30 μm)	BEAS‐2B and THP‐1a Submerged (24 h) TGF‐β1, PDGF‐AA, and IL‐1β	C57BL/6 mice Oropharyngeal aspiration (24 h and 21 days) TGF‐β1, PDGF‐AA, and IL‐1β in BALF and histology	Better dispersed MWCNT induced more TGF‐β1, PDGF‐AA, and IL‐1β in cells and animals in BALF, which was confirmed by the fibrogenic signs in lung tissue. Results on profibrogenic responses in vitro and in vivo effects showed good agreement
Han et al. (2012)	TiO_2_ (8 different samples with different size 3–104 nm)	Rat lung epithelial cells line (R3/1) Submerged (24 h) Protein oxidation (carbonyls) and cytotoxicity (LDH)	Male Fisher 344 Intratracheal instillation (24 h) Neutrophil influx in BALF	In vitro‐in vivo correlation with steepest slope analysis method, only after doses were normalized by surface area; *r*_*s*_ = 0.93, *p* < 0.01 (protein oxidation versus PMN) and *r*_*s*_ = 0.88, *p* < 0.01 (LDH versus PMN)
Zhang et al. (2012)	CuO, CeO_2_, Co_3_O_4_, CoO, Cr_2_O_3_, Fe_2_O_3_, Fe_3_O_4_, HfO_2_, In_2_O_3_, Mn_2_O_3_, NiO, Ni_2_O_3_, Y_2_O_3_, ZrO_2_	BEAS‐2B and RAW264.7 Submerged (24 h) MTS, ATP, LDH, high‐throughput screening for oxidative stress biomarkers	C57BL/6 mice Oropharyngeal aspiration (40 h) Neutrophil influx in BALF, MCP‐1, IL‐6 in BALF	Correlation between cytotoxicity (combined MTS, ATP, LDH) and neutrophil influx (*r*_*p*_ = 0.76, *p* < 0.01) (calculated from mean values reported in article) and oxidative stress biomarker (heme oxygenase 1) in lung tissue (Co_3_O_4_, Cr_2_O_3_, Ni_2_O_3_, Mn_2_O_3_, and CoO)
Cho et al. (2013)	TiO_2_, CeO_2_, SiO_2_, NiO, Co_3_O_4_, Cr_2_O_3_, CuO, ZnO and carbon black	16‐HBE, A549, THP‐1 monocytes, THP‐1a, primary PBMC, A549 + THP‐1a Submerged (24 h) IL‐8, TNF‐α, LDH, and hemolysis	Female Wistar rats Intratracheal instillation (24 h) Neutrophil influx in BALF	Concordance between pulmonary inflammation in lung in vivo and cytotoxicity in PBMC was 89% (11% false negative), followed by primary cultured alveolar macrophages (78% concordance, 11% false negative, and 11% false positive)
Li et al. (2013)	Pristine and f‐MWCNT; Carboxylate (COOH), polyethylene glycol (PEG), amine (NH2), sidewall amine (sw‐NH2), and polyetherimide (PEI)‐modified MWCNTs	BEAS‐2B and THP‐1a cells Submerged (24 h) IL‐1β,TGF‐β1, and PDGF‐AA	C57Bl/6 male mice Oropharyngeal aspiration (40 h) Collagen deposition in lung tissue (Sircol assay, Trichrome staining), TGF‐b, IL‐1b, TGF‐b1, and PDGF‐AA in BALF	PEI‐MWCNTs induced the highest IL‐1β, TGFβ, and PDGF‐AA production in vitro and in vivo, and more pronounced collagen deposition than other MWCNT. MWCNTs with the lowest hazard ranking in vitro (i.e., AF and COOH) did not increase the presence of collagen in lung tissue
Pal et al. (2015)	TiO_2_ P25, CeO_2_, Ni, Ag, CB Printex 90	THP‐1a Submerged (24 h) Inflammation (IL‐8), cytotoxicity (LDH)	Male Fischer rats (F344/DuCrl) Intratracheal instillation (24 h) PMN cells in the BALF	Correlation between cell dell death in THP‐1a and PMN in rats (deposited doses: *r* = 0.98; administered doses: *r* = 0.80, linear regression). Correlations were not reported for IL‐8. However, based on results in the publication, correlation and linear regression coefficient is 0.10, which is not statistically significant
Ma et al. (2015)	Graphene oxides (GO) with differential sizes (small: 50–350 nm; large: 750–1300 nm)	J774.A1, THP‐1a Submerged (24 h) IL‐6, TNF‐a, ROS production (DCFH‐DA)	BALB/c mice Oropharyngeal aspiration (72 h) PMN cells in BALF	Large GO had higher proinflammatory response in vitro (TNF‐a and IL‐6 production in J774.A1 cells; *TNF‐a* expression in THP‐1a cells) and in vivo (total cells in BALF). Similar ROS production by small and large GOs in J774.A1 cells
Wiemann et al. (2016)	CeO_2_, Fe_2_O_3_, TiO_2_, ZrO_2_ (pristine and surface modified), ZnO NMs, amorphous SiO_2_ (pristine and surface modified), graphite nanoplatelets, 2 nanosized organic pigments, quartz DQ12, and corundum	Rat NR8383 alveolar macrophage Submerged (16 h) LDH, GLU, TNF‐α, and H_2_O_2_ release	Wistar rat Inhalation (6 h exposure/day, 5 consecutive days) NOAEC derived from PMN, LDH, cytokines	The in vitro NR8383 alveolar macrophage assay allows distinguishing active from passive nanomaterials (concordance = 95%)
Wiemann et al. (2018)	SiO_2_ (amorphous, 15 or 55 nm)	Rat NR8383 alveolar macrophage Submerged (16 h) LDH	Wistar rats Intratracheal instillation of 15 and 55 nm SiO_2_ and inhalation of 15 nm SiO_2_ (3 days) Cell infiltrates and LDH in BALF	Higher response in terms of LDH release with 15 nm particles compared with 55 nm particles in both cells and animals (the authors tested also TNF‐α, β‐glucuronidase, H_2_O_2_ release)
Loret et al. (2018)	TiO_2_ (NM‐100, NM‐101, and NM‐105)	A549 and THP‐1a in co‐culture ALI, submerged (24 h) IL‐1β, IL‐8, IL‐6, TNF‐α, LDH	Wistar rats Intratracheal instillation (24 h) LDH and proinflammatory mediators (IL‐1β, IL‐8, IL‐6, and TNF‐α) in BALF	Same ranking based on cytotoxicity and 20% increase in proinflammatory response in cell cultures and animals, using surface area as dose metric
Cui et al. (2019)	ZnO, SiO_2_, TiO_2_, and CeO_2_	BEAS‐2B Submerged (24 h) Metabolomics	C57BL/6 mice Oropharyngeal aspiration (40 h) Levels of metabolites	Similar response on levels of metabolites after exposure to ENMs in vitro and in vivo (the study also assessed proinflammatory responses in vitro [e.g., IL6 and IL8] and in vivo [e.g., neutrophils in BALF]. The responses do not appear to be congruent, which was not described in the article
Di Ianni, Erdem, et al. (2021)	MWCNTs (length: 443–573 nm; diameter: 11–74 nm)	A549, THP‐1a, A549:THP‐1a:WI‐38 Submerged, ALI (6, 24 h) IL‐8, DNA damage	C57Bl/6 mice Intratracheal instillation (24 h) neutrophil influx in BALF and DNA damage in BALF cells and lung tissue	Correlation between IL8 secretion in A549 cells (*r* = 0.92 and 0.95 for A549 (6 and 24 h, respectively) versus BAL cells, and *r* = 0.96 for THP‐1a (both 6 and 24 h) versus BAL cells). Concordant DNA damage between THP‐1 cells and BAL cells (83%)
Di Ianni, Møller, et al. (2021)	Pristine and surface modified nanoclays; graphene oxide and reduced‐graphene oxide	A549, THP‐1a Submerged (6, 24 h) IL‐8, DNA damage	C57Bl/6 mice Intratracheal instillation (24 h, 3 days) neutrophil influx in BALF and DNA damage in BALF cells and lung tissue	Correlation between IL8 secretion in A549 cells versus neutrophils; *r* = 0.96 (*p* < 0.01) and *r* = 0.97 (*p* < 0.01) at 6 and 24 h, respectively; THP‐1a cells versus neutrophils: *r* = 0.93 (*p* = 0.02) and *r* = 0.92 (*p* = 0.02) at 6 and 24 h, respectively Concordant DNA damage between THP‐1a cells and BAL cells 1 (60%) and 3 days (80%) postexposure
Karkossa et al. (2021)	SiO_2__15 Amino, SiO_2__15 unmod, SiO_2__7, SiO_2__40, TiO_2__NM105	RLE‐6TN (rat alveolar epithelial cells) NR8383 (rat alveolar macrophages) Submerged conditions (24 h) Metabolomic an proteomic changes in oxidative stress	Wistar rat Short‐term inhalation (6 h/day over 5 days, euthanized right after 5th day or after 21 days) Intratracheal instillation (3 and 6 days) Metabolomic and proteomic changes in oxidative stress	different changes of markers in all the models; congruent classification of “active” and “passive” materials in vitro and in vivo, based on significantly changed signatures in exposed groups compared with controls
Fujita et al. (2022)	Three carbon fibers with different diameters and length: SCF1 diameter: 259 nm length: 12 μm SCF2 diameter: 248 nm length: 7 μm SCF3 diameter: 183 nm; length: 14 μm	NR8383 rat alveolar macrophages Submerged conditions (24 h) Cell viability, MIP‐1α, IL‐18, TNF‐α	Sprague‐Dawley rat Intratracheal instillation (1, 3, 7, 30, 90, and 180 days) BALF analysis and histology	In vitro, the ranking was observed to be SCF3 > SCF1 > SCF2, based on cell death, MIP‐1a, IL‐18, and TNF‐α. In vivo, the same ranking was observed based on neutrophil influx, eosinophils, total protein, and LDH in BALF

Abbreviation: BALF, bronco‐alveolar lavage fluid.

The review does not systematically address factors for differences in toxicological effects of different ENMs. Rather, we assume the ENMs in each study have been selected to introduce a gradient in hazardous properties for the purpose of comparing toxicological responses in cells and animals. Thus, one study may have selected material A and B, and another study material C and D; the ENMs cannot be compared, but the difference between them can. In this respect, it does not affect the analysis that material A–D have different mediators of toxicity such as solubility, particle surface area, reactive oxygen species (ROS) production potential, and frustrated phagocytosis. In fact, it is far more concerning if researchers have selected ENMs that are too similar, because it might make it difficult to obtain a wide difference in toxicological responses in both cell cultures and animals. The studies described here used different cell types, mostly epithelial cells and macrophages, including primary cells and, in the majority of the studies, cancer cell lines. Of these studies, only three compared responses in the ALI system and lung tissue of animals. We have segregated the studies according to the level of complexity of the in vitro cell models (Di Ianni, Erdem, et al., 2021; Loret et al., 2018). Monoculture of cell lines in submerged conditions is regarded as a “simple” test system (lowest level). Test systems using co‐cultures, primary cells, or ALI are more complex than the simple monoculture system. The most complex text system encompass co‐cultures of primary cells in ALI. Table 2 outlines the different classes of complexity and the belonging of the studies in each of these categories.

**TABLE 2:** Assignment of class of complexity to the cell‐culture models used in in vitro*–*in vivo comparison of toxicological responses

Submerged/ALI	Mono‐/co‐cultures	Cell line/primary cell	Total	Class complexity	References
0	0	0	0	A	Rushton et al. (2010); Wang et al. (2011); Han et al. (2012); Zhang et al. (2012); Cho et al. (2013); Li et al. (2013); Ma et al. (2015); Pal et al. (2015); Wiemann et al. (2016); Loret et al. (2018); Wiemann et al. (2018); Cui et al. (2019); Di Ianni, Erdem, et al. (2021); Di Ianni, Møller, et al. (2021); Karkossa et al. (2021); Fujita et al. (2022)
1	0	0	1	B	Li et al. (2013)^a^
0	1	0	1	B	Sayes et al. (2007); Warheit et al. (2009); Loret et al. (2018)
0	0	1	1	B	No study
1	1	0	2	C	Di Ianni, Erdem, et al. (2021); Loret et al. (2018)
1	0	1	2	C	No study
0	1	1	2	C	No study
1	1	1	3	D	No study

*Note*: “0” is given to models in “Submerged conditions,” “Monocultures,” or “Cell lines,” whereas “1” is given to models that were in “ALI,” “Co‐cultures,” or “Primary cells.” The total (0–3) leads to complexity class (A–D) with class A being the least complex and D the most complex.

^a^ Li et al. (2013) mentions the use of BEAS‐2B and THP‐1a co‐culture, but the results are not specifically described.

The in vivo data were generated from exposure of mice or rats via inhalation, oropharyngeal aspiration, and intratracheal instillation. The endpoints correlated were related to cytotoxicity, inflammation, and oxidative stress, and only two studies included DNA damage. The original papers and the analyses in the review use either correlations or concordance as tools to compare the relationship between responses in cell cultures and animals. The meaning and use of these and other terms are described below.

### 2.1 Correlation

Test using continuous data on the predictor (endpoint in vitro) and outcome scale (endpoint in vivo). The data may come from effects at fixed doses in cells or animals, or from the slopes of individual dose–response curves in cell cultures and animal models. The results are usually assessed by correlation analysis using assumption of a qualitative (e.g., Spearman correlation test) or quantitative (e.g., linear regression or Pearson correlation test) relationship between responses in cell cultures and animals.

### 2.2 Concordance

Test that determines the proportion of correct outcomes of a test method (i.e., correctly identified positives and negatives). The analysis also generates information about positive predictivity, negative predictivity, sensitivity, and specificity. This is based on binary segregation of results into level of responses according to predefined criteria such as a certain fold‐increase or statistical significance. Concordance is often used interchangeably with “accuracy,” but the latter term is also used to describe the closeness of agreement between test results and accepted reference values. There is not a threshold of acceptable concordance of a test in genetic toxicology. However, the Ames test—the most used regulatory test for genotoxicity—has 70% concordance (Zeiger, 2019). The concordance of carcinogenicity in mice and rats has been shown to be 75% (Gold et al., 1991).

### 2.3 Dose

In traditional toxicology dose refers to the mass of a poisonous agent relative to the mass of the biological system (e.g., mg/kg bodyweight), whereas concentration refers to the mass relative to the volume. Most studies on ENMs are designed to expose cells or animals to fixed masses of particles (i.e., the nominator is a mass unit). The mass is often transformed to surface area, using the specific surface area of particles (i.e., nominator) or surface area of the cell culture dish or animal epithelial area (i.e., denominator). In the present article, we refer to the exposure level as dose to keep it simple. Nevertheless, it should be emphasized that the true dose in cell culture systems is uncertain as it depends on the sedimentation rate of particles.

### 2.4 Potency

Certain studies have used potency to rank the hazard of ENMs (Breznan et al., 2016; Loret et al., 2018). Potency is the exposure of a compound in terms of the dose that is needed to produce a predefined effect (e.g., low dose is equivalent to high potency).

## 3 DISCUSSION

### 3.1 Can cell cultures predict pulmonary toxicity in animals?

A number of studies on ENMs have assessed whether data from in vitro assays predict positive (toxic) or negative (nontoxic) effects in exposed animals. This distinction between toxic and nontoxic materials derives in most studies from a qualitative assessment between in vitro and in vivo responses. Table 3 summarizes the studies that have been used to assess the predictivity of cell culture studies on outcomes in animal models. It is not possible to apply the same analysis of the association or ranking of hazards of ENMs between results from in vitro and in vivo studies because the studies have had different experimental designs. We have segregated the studies into experiments that have resulted in formal analysis of the predictivity or correlation between in vitro and in vivo responses, and those that have used a descriptive approach. Cho et al. (2013) assessed responses of in vitro assays with in vivo acute lung inflammogenicity for a set of metal oxide ENMs with a broad gradient in physicochemical properties. From the in vitro assays, an ENM was classified as “positive” if it caused any statistically significant increase at any dose, otherwise it was classified as “negative” (i.e., no effect) (Cho et al., 2013). For in vivo experiments, statistically significant increases in the number of total granulocytes in bronchoalveolar lavage fluid (BALF) compared with vehicle control were regarded as “positive.” The authors made also a comparison of responses at a fixed dose in the in vitro assays (30 cm^2^/ml, i.e., the only overlapping dose used for all the particles except for CuO, where 3 cm^2^/ml was the highest dose; Cho et al., 2013). From the comparison of statistically significant effects at any dose, hemolysis assay appeared to be the most predictive (concordance = 100%), followed by cytotoxicity in differentiated peripheral blood mononuclear cells (PBMCs; 78% concordance, 11% [1/9] false negativity, and 11% [1/9] false positivity), while other assays showed poorer concordance (≤67%). In contrast, with the comparison at equal dose, cytotoxicity in PBMCs was the most predictive, with 89% (8/9) concordance, 11% (1/9) false negativity, and 0% (0/9) false positivity, followed by cytotoxicity using primary cultured alveolar macrophages (78% [7/9] concordance, 11% [1/9] false negativity, and 11% [1/9] false positivity), while all other assays showed a poorer association (concordance ≤67%). Similarly, Wiemann et al. assessed the concordance in the effects between in vitro exposure in macrophages (NR8383 rat cells) and rat inhalation exposure for a set of 20 ENMs. The authors defined the no‐observed‐adverse‐effect concentration (NOAEC) of <10 mg/m^3^ as threshold value indicating ENMs to be positive in vivo (inflammatory reactions defined by hematology, BALF evaluation, and/or lung histopathology), whereas NMs with NOAECs of ≥10 mg/m^3^ were categorized as being negative. For the in vitro assessment, the authors tested the ENMs in macrophages and quantified lactate dehydrogenase (LDH), β‐glucoronidase (GLU), tumor necrosis factor alpha (TNF‐α), and ROS production. The authors then assessed the lowest observable effect concentration (LOEC) for each ENM, and normalized it by the ENM Brunauer–Emmett–Teller (BET) surface area. ENMs were classified as positive if the in vitro LOECs were below 6000 mm^2^/ml for at least two of the four in vitro endpoints (e.g., elevated LDH and TNF‐α). With this setup, the in vitro responses predicted the inflammation in vivo with 95% concordance, 91% specificity, and a 100% sensitivity (Wiemann et al., 2016). Karkossa et al. (2021) investigated a group of metal oxide nanomaterials that included four SiO_2_ NMs and one TiO_2_. The authors tested with metabolomic and proteomic changes in the pathways involved in the oxidative stress in rat alveolar epithelial cells, macrophages, and rat lung. The authors observed that the changes of markers were different in all the models; however, the classification of “active” or “passive,” based on significantly changed signatures in exposed groups compared with controls, led to the congruent classifications between in vitro and in vivo (Karkossa et al., 2021).

**TABLE 3:** Summary of studies that have assessed the predictivity of cell culture studies on outcomes in animal models

Author	Analysis	No. of ENMs	Response (biomarker)	Key outcome
Cho et al. (2013)	Statistical	9	Different in vitro effects (cytotoxicity, proinflammatory cytokines, and hemolysis) versus inflammation (in vivo)	100% concordance (hemolysis) 59% concordance (cytotoxicity)^a^ 68% concordance (cytokines)^a^
Wiemann et al. (2016)	Statistical	15	Different in vitro effects (cytotoxicity, proinflammatory cytokines and ROS production) versus inflammation (in vivo)	95% concordance^b^
Di Ianni, Erdem, et al. (2021)	Statistical	8	Genotoxicity	83% concordance (THP‐1a versus BAL cells) 32% concordance (THP‐1a versus lungs) 50% concordance (A549 versus BAL cells 14% concordance (A549 versus lungs)
Di Ianni, Møller, et al. (2021)	Statistical	5	Genotoxicity	60% concordance (THP‐1a versus BAL cells) 20% concordance (A549 versus BAL cells)
Wiemann et al. (2018)	Descriptive	2	Cytotoxicity (LDH activity)	Same response in vitro and in vivo (higher LDH activity by 15 nm as compared with 55 nm SiO_2_)
Ma et al. (2015)	Descriptive	2	Cytotoxicity and inflammation	Same response in vitro and in vivo (“large” graphene sheets more hazardous than “small” sheets for both cytotoxicity and inflammation biomarkers)
Wang et al. (2011)	Descriptive	2	Profibrotic responses	Same hazard ranking of ENMs in vitro (profibrotic mediators) and in vivo lung (profibrotic mediators and collagen staining)
Li et al. (2013)	Descriptive	4	Proinflammatory cytokines (cells) versus fibrosis (animals)	Same hazard ranking of ENMs in vitro cytokine production and in vivo lung collagen staining
Cui et al. (2019)	Descriptive	4	Metabolites	Comparable metabolomics signature in vitro and in vivo

^a^ Mean values of concordances in different cells and proinflammatory cytokines.

^b^ In vitro hazard threshold is based on combined results from cytotoxicity, cytokine release, and ROS production assays.

Only one research group has assessed the predictivity of in vitro genotoxicity results on the same endpoint in animal lungs and BALF cells. The categorical classification of ENMs as genotoxic or nongenotoxic agent was based on DNA strand breaks, measured by the standard alkaline comet assay in species exposed to multiwalled carbon nanotubes (MWCNT), graphene oxide (GO), and clay nanomaterials (Di Ianni, Erdem, et al., 2021; Di Ianni, Møller, et al., 2021). A scoring system was developed based on standard deviation fold increase units. The categories were defined within control groups, to which the exposed groups for each ENM were compared. The comparison resulted in good concordance in DNA damage of MWCNT between activated THP‐1 macrophages (THP‐1a) and BALF cells of mice (83%, 5/6 correctly assigned), whereas there was less concordance with genotoxic responses in lung tissue (33%, 2/6). Test results for A549 cells were less concordant with the response in BALF cells (concordance = 50%) and lung tissue (concordance = 14%; Di Ianni, Erdem, et al., 2021). Using the same scoring system, there was good concordance in DNA damage caused by flake ENMs (nanoclays, GO and rGO) in THP‐1a cells (4/5) and BALF cells of exposed mice groups at day 3 post‐exposure (4/5), whereas DNA damage in A549 cells (2/5) was less concordant with the DNA damage in BALF cells (Di Ianni, Møller, et al., 2021).

Some studies have assessed how the variation in one variable/predictor, for example, ENMs size or other physicochemical property, affects the outcomes quantified in vitro and in vivo. This generates information about the equivalence of the test result relative to the physico‐chemical characteristic between the two test systems. Wiemann et al. (2018) showed for 15 and 55 nm SiO_2_ the levels of cytotoxicity were size dependent and the dependency was concordant in vitro and in vivo (Wiemann et al., 2018). Similarly, Ma et al. (2015) showed that proinflammatory responses of GO increased with increasing lateral size of the particles in both cell cultures and animals. Wang et al. (2011) showed that experiments on well‐dispersed MWCNTs led to equivalent conclusions on hazard of fibrosis in vitro and in vivo, whereas experiments using poorly dispersed MWCNTs produced inconsistent responses. When MWCNT were well dispersed in exposure medium, these induced a larger activation of fibrotic biomarkers in epithelial cells and macrophages, which was found to be consistent with the extent of fibrosis detected in lungs in vivo (Wang et al., 2011). Li et al. (2013) assessed in vitro and in vivo the profibrotic changes following exposure to a group of pristine and surface modified MWCNT. The authors identified cationic MWCNT (polyetherimide‐modified MWCNT) to cause strongest effects in terms of platelet‐derived growth factor AA (PDGF‐AA), tissue growth factor β1 and interleukin (IL) 1β in vitro (BEAS‐2B and THP‐1a cells) and in vivo (same mediators in BALF or collagen staining in lung tissue), compared with other MWCNT, and concluded that the proinflammatory changes were comparable between the two models.

A similar qualitative approach was used to identify the biomarkers that are expressed in both in vitro and in vivo models following ENMs exposure. Cui et al. (2019) found comparable responses in terms of levels of metabolites in bronchial epithelial cells vitro and lung tissue of mice following exposure to a set of metal oxide ENMs, suggesting that the quantification of some metabolites in vitro could be used to predict the release of metabolites in vivo. The same study also assessed proinflammatory responses in cells and animal lungs, but there appeared to be no consistency between the responses seen in vitro and in vivo.

Taken together, binary classification of ENMs toxicity in vitro and in vivo could allow determining whether in vivo testing is necessary for hazard assessment. Results may also be used to group ENMs by biological activity.

### 3.2 Does the magnitude of response in cell cultures correspond to the magnitude of pulmonary toxicity in animals?

**FIGURE 2:**
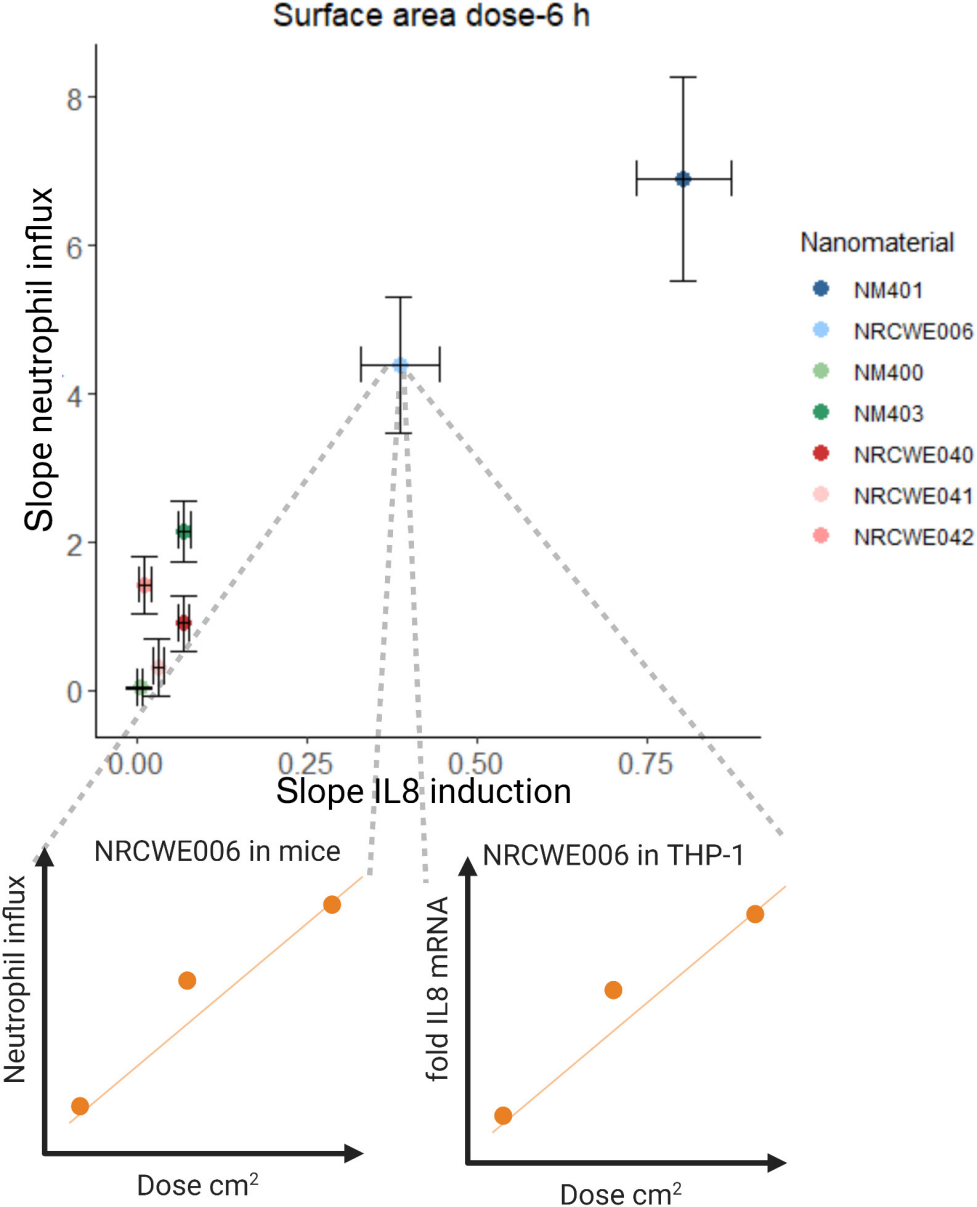
Example of correlation of ENM‐induced effects by use of dose–response slope in cell culture and animal experiments. MWCNT (three surface area doses) were tested in THP‐1a cells and in mice (bottom). The slopes (regression coefficients) of the different MWCNTs were subsequently analyzed by linear regression analyses (Pearson correlation test) of the in vitro and in vivo results (top). Adapted under terms of Creative Commons Attribution 4.0 International License (Di Ianni, Erdem, et al., 2021)

**FIGURE 3:**
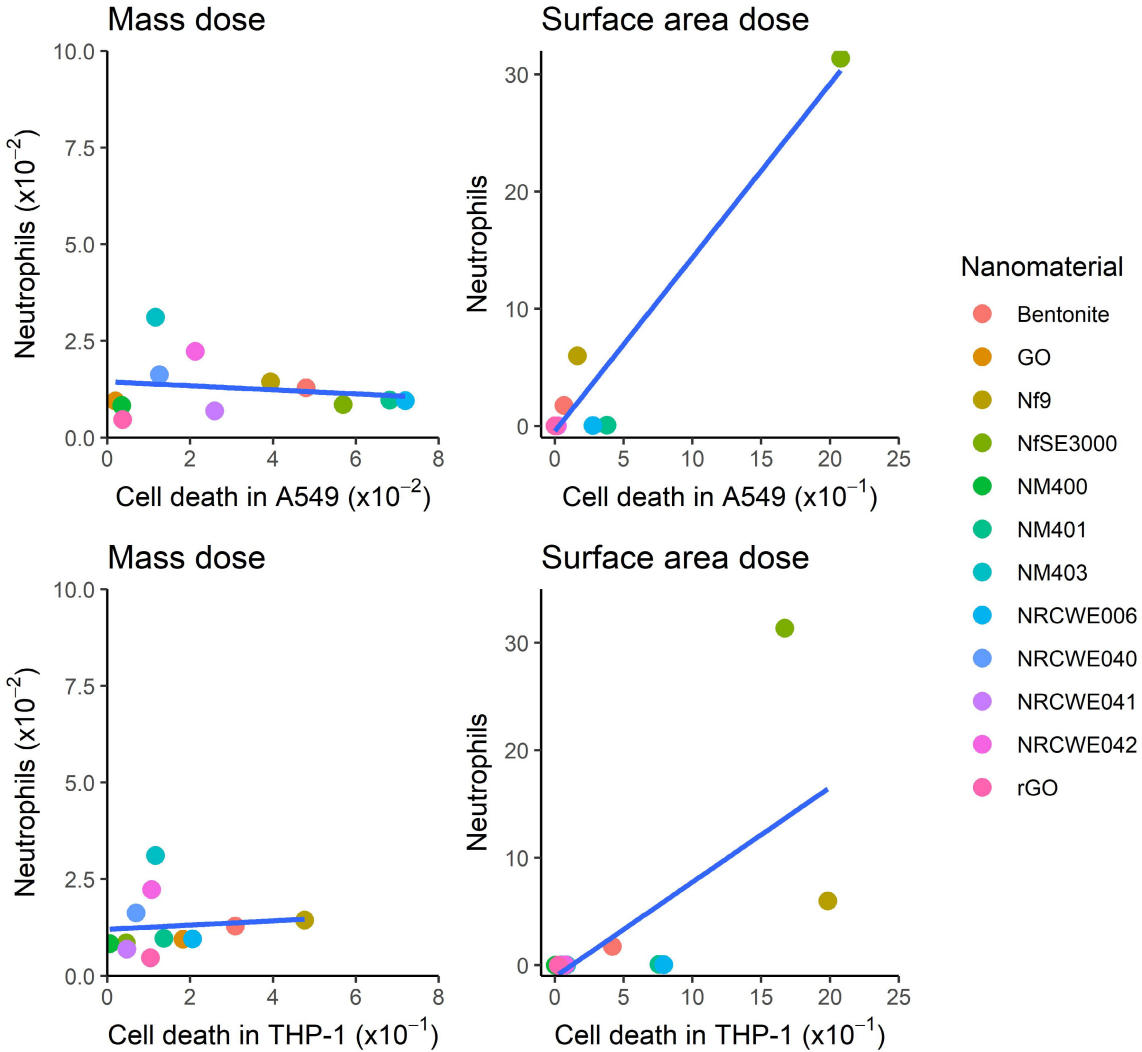
In vitro–in vivo correlations of cell death in A549 and activated THP‐1 cells (i.e., macrophage‐like) and neutrophil influx following exposure to MWCNT, nanoclays, and graphene oxide. Results on the axis are slopes from linear regression analysis in cells and animals (predictor: Cell death in cells per mass or surface dose; dependent variable: Neutrophils in BALF per mass or surface dose). The correlations were assessed with doses expressed as mass, or normalized against ENM surface area. In vitro correlation with neutrophil influx on mass basis: *r* = −0.18 and *p* = 0.57 with A549, *r* = 0.09 and *p* = 0.76 with THP‐1a. In vitro correlation with neutrophil influx on surface area basis: *r* = 0.97 with *p* < 0.001 for A549, *r* = 0.67 with *p* = 0.02 for THP‐1a. The correlations have been calculated from previously published data (Di Ianni, Erdem, et al., 2021; Di Ianni, Møller, et al., 2021). Figure created with RStudio using “ggplot2” package (Valero‐Mora, 2010)

The magnitude of ENMs‐induced effects in cells and animals have been proven valuable for the in vitro–in vivo correlation assessment. This type of assessment is based on comparisons of dose–response relationships for groups of ENMs, whereby the difference in the slopes is used for hazard rankings (Figure 2). Rushton et al. (2010) compared the steepest slopes of a set of ENMs, because the steepest part of a nonlinear dose–response curve is the response that is induced by intermediate doses and therefore is most sensitive to predict the variation in response. The responses were normalized by the ENM surface area, resulting in a response per unit surface area, as the response‐metric. Linear fit models were used to correlate the surface area‐normalized responses in vitro with in vivo. All in vitro responses in terms of ROS production or expression of IL‐8, using a luciferase reporter gene, correlated significantly with in vivo results (*r*_*s*_ = 0.94) (Rushton et al., 2010). Sayes et al. observed no correlation between in vitro and in vivo responses induced by a set of ENMs, using mass‐dose metric (Sayes et al., 2007). Subsequently, Rushton et al. (2010) used the steepest slope‐approach with the same dataset and showed good correlation after normalizing the response by use of the ENMs surface area. By using the same approach with slopes, Han et al. (2012) showed that in vitro and in vivo responses following exposure to TiO_2_ ENM with different size and crystallinity were well correlated (*r*_*s*_ = 0.93 and 0.88 for correlations between protein oxidation or LDH release in cells and influx of polymorphonuclear cells [PMNs] in BALF in vivo, respectively), suggesting that using the approach of steepest slope analysis offers a superior method to correlate in vitro with in vivo results of ENM toxicity and for ranking their toxic potency. Similarly, slopes of *IL‐8* expression in A549 and THP‐1a cells after exposure to multiple MWCNT, GO and nanoclay were significantly correlated with inflammogenicity of the same materials in mice in terms of neutrophil influx (*r*_*s*_‐values ≥0.92) after doses were normalized by use of ENMs BET surface area (Di Ianni, Erdem, et al., 2021; Di Ianni, Møller, et al., 2021). In the same dataset, we have observed positive correlation between cell death in A549 cells and THP‐1a cells and neutrophil influx in mice (Figure 3). More recently, Fujita et al. tested the pulmonary toxicity of three fibers with different diameters and length (SCF1, SCF2, and SCF3). In vitro, the ranking was observed to be SCF3 > SCF1 > SCF2, based on cell death, MIP‐1a, IL‐18, and TNF‐a. In vivo, the same ranking was observed based on neutrophil influx, eosinophils, total protein, and LDH in BALF. This demonstrated similar potential hazard effects in vitro and in vivo, namely the hazard increases with reduction of diameter and increase of length. It is important to note though that the evaluation included only three materials, which minimizes the chance of rejecting a conclusion of high concordance in hazard ranking in vitro and in vivo (Fujita et al., 2022).

Zhang et al. (2012) tested a set of metal oxide ENMs and demonstrated a concordant ranking of effect in vitro and in vivo by using normalized toxicological size effects. The in vivo effect size was defined by a T‐statistic value that describes the difference between the mean neutrophil cell counts of metal oxide‐exposed versus nonexposed animals. The in vitro effect size was defined as the predicted probability of cytotoxicity according to a regression model that assigns a number between 0 and 1 for each ENM, with “1” meaning 100% confidence of a cytotoxic effect (or IL‐6 and monocyte chemoattractant protein‐1 [MCP‐1] upregulation) and “0” meaning no chance. In this comparative analysis, ENMs with an in vitro toxicological probability >50% were statistically more likely to induce a high neutrophil influx in BALF. The in vitro toxicity response (cytotoxicity, MCP‐1, and IL‐6) correlated well with the T‐statistic value of the in vivo inflammation data (*r*_*p*_ > 0.76) as well as with oxidative stress biomarkers (i.e., heme oxygenase 1) in lung tissue (Zhang et al., 2012).

Regression analyses have been used for in vitro hazard assessment. Pal et al. investigated the implications of the dosimetry on the hazard ranking, and demonstrated that correlating the slopes of a group of ENMs with “effective doses” instead of administered doses improved the correlation between in vivo lung inflammation and in vitro cell viability, with the regression coefficient (*r*_linear regression_) increasing from 0.80 to 0.98. However, a closer inspection of the results shows that the inflammatory potency in cells (based on highest induction of IL‐8) and PMNs in BALF did not correlate (*r*_*s*_ = 0.1, *p* = 0.87, results abstracted from figures in the publication). This highlights the uncertainty in generalizing associations between specific outcomes to hazards of ENMs. Loret et al. (2018) used benchmark dose–response modeling to determine ENMs (3 TiO_2_ and 1 CeO_2_) dose intervals related to increase in proinflammatory mediator levels in vitro and in vivo. An effect of 20% increase of proinflammatory mediator level compared with nonexposed controls was used as critical value, and the intervals were defined as Benchmark Dose Lower confidence limit (BMDL) and the Benchmark Dose Upper confidence limit (BMDU) of the interval for a 90% confidence. The authors compared the intervals defined by different dose metrics. Although there were similarities in the ENMs ranking using in vivo and in vitro approaches, the dataset was not sufficiently large to provide firm ranking comparisons (Loret et al., 2018). It might be that including more potent ENMs would have enlarged the gradient in the toxicity and thereby improved the hazard ranking.

In summary, these studies using rankings or correlations on quantitative scale demonstrate that the magnitude of effects induced in vitro can resemble the responses in vivo, however the in vitro–in vivo comparisons need a gradient in the toxicity to reveal strong association of slopes or rankings.

### 3.3 Are advanced cell culture models better predictors of pulmonary toxicity in animals as compared with monocultures?

Inhaled nanoparticles deposit in different regions of the lungs depending on their size. Those in the smaller size range can deposit in the alveoli (Oberdörster et al., 2005). Different cell models can be implemented to mimic the lungs in different regions. Epithelial cells and macrophages are normally used in 2D monoculture and sometimes they are combined in co‐culture to increase complexity due to cell–cell communication. To further increase the complexity, fibroblasts have been included in co‐culture systems (Table 1). The level of complexity is also defined by the origin of the cells (e.g., primary or cancer cell line), as well as exposure system (submerged or ALI, discussed in the next section). We have arbitrarily defined categories of complexity, which are outlined in Table 2. Increasing the complexity of the cell models should better mimic the cellular microenvironment of the lungs and better predict the occurrence of adverse effects in vivo. As shown in Table 1, different cell culture models have been implemented in studies aiming at in vitro–in vivo comparisons of ENMs‐induced oxidative stress, inflammation, and genotoxicity. A large number of studies shows positively correlated oxidative stress and inflammation between bronchial (BEAS‐2B or 16‐HBE) and alveolar (A549) epithelial cell lines as well as macrophage cell lines (human, THP‐1a; mouse, RAW264.7, and J774A.1). Two studies reported also good concordance of genotoxic effects between THP‐1a cells and BALF cells from mice lungs, whereas the concordance was poor for genotoxicity in A549 cells and mice lung tissue (Di Ianni, Erdem, et al., 2021; Di Ianni, Møller, et al., 2021). In contrast, there are very few studies showing positive correlation of the responses to ENM exposure in animal lungs and primary cells (human primary blood monocytes derived macrophages, mouse, and rat macrophages) (Cho et al., 2013; Jing et al., 2015), while others were less successful (Sayes et al., 2007; Warheit et al., 2009). The dataset generated in Sayes et al. (2007) was used by Rushton et al. (2010) to assess correlations with the steepest slope method, which resulted in good correlations between macrophage inflammatory protein‐2 (MIP‐2) in alveolar macrophages (AMs) and neutrophil influx (*r*_*s*_ = 0.96, *p* = 0.04), or LDH in L2‐AMs co‐culture and neutrophil influx (*r*_*s*_ = 0.96, *p* = 0.002), when doses were normalized by surface area (Rushton et al., 2010). However, Sayes et al. (2007) reported also other endpoints including TNF‐α, IL‐6 and cytotoxicity (i.e., MTT assay), but these endpoints were not included in this later analysis. Using cell lines might be beneficial in performing high‐throughput screening of toxicity of libraries of ENMs and correlate to in vivo responses.

The combination of different cell types is proposed to be pivotal to increase the physiological relevance of in vitro models. However, of studies reported in Table 1, only Loret et al. (2018) show the same ranking of potency between co‐cultures of A549 and THP‐1a exposed in the ALI system, yet they did not compare the effects of the co‐culture to a monoculture. In line with this, Di Ianni, Erdem, et al. (2021) showed that the co‐culture of A549 and THP‐1a cells exposed to MWCNT resulted in dose–response relationships that were comparable to those obtained in monocultures. Two other studies did not show consistency in proinflammation responses between co‐cultures an in vivo (Sayes et al., 2007; Warheit et al., 2009). Rushton et al., 2010 used results by Sayes et al. (2007) and showed that normalized concentrations by use of ENM surface area produced equally good correlations between monoculture or co‐culture, and neutrophil influx. To date, the limited number of studies showing positive in vitro–in vivo correlations with complex cell models hinders the conclusions regarding whether co‐cultures better predict ENMs adverse effects in vivo.

The system in which cultured cells are exposed has been focus of discussions in the nanotoxicology field. Most of the in vitro toxicity tests employ exposure of cells in submerged conditions, whereby ENMs are administered from a particle suspension. Proliferating cells like alveolar epithelial cells in submerged condition covered by liquid are far from their real physiological microenvironment, as these cells grow at a gas phase and covered by a thin layer of lung surfactant (Parra & Pérez‐gil, 2015). When biological effects of ENMs exposure are tested in submerged conditions, ENMs are dispersed in cell culture medium and administered to the cells, which are adherent to the bottom of the well. After administration, the dispersed ENMs sediment and reach adherent cells over time. The use of inserts (porous membranes) allows for culturing cells in ALI systems and for exposure to aerosolized ENMs. This better mimics the microenvironment of the lung epithelial surface: cells are in contact with medium at the basolateral side of the insert, while exposed to an aerosol at the apical side. Therefore, it has been argued that cells exposed to ENMs in the ALI system might be more predictive of the effects in the lungs. When considering the studies that have to date compared ENM toxicity in vitro with in vivo we have found that the largest majority obtained good correlations with exposure in submerged conditions (Table 1 and Figure 1). One study reported the exposure of cell culture models in the ALI to elicit comparable results to the responses observed in vivo (Loret et al., 2018). Nevertheless, it has been shown that exposure in the ALI of low‐density materials like MWCNT has methodological limitations including nebulization of highly concentrated suspensions of materials, and the inflammatory response by cell exposure to MWCNT in the ALI system is comparable to the effective doses (quantified by thermogravimetric analysis) in submerged conditions (Di Ianni, Erdem, et al., 2021). In these two studies (Di Ianni, Erdem, et al., 2021; Loret et al., 2018), the ALI exposure used quantification of deposited doses by quartz‐crystal microbalance (QCM), which was shown to be a sensitive and accurate device for real‐time dosimetry of the cell‐delivered particle dose (Ding et al., 2020). The quantification of “real,” sedimented doses in submerged conditions, determined by In vitro Sedimentation, Diffusion, and Dosimetry (ISDD) model, has been shown to improve hazard assessment of ENMs in vitro and thereby the correlations with hazard rankings in vivo (Pal et al., 2015). Similarly, Loret et al. (2018) found an improvement in the dose‐effect estimates when adopting the ISDD model, however non‐significant.

## 4 PERSPECTIVES

This review aimed at collecting and discussing studies that have compared toxicity responses in cell cultures and animal lungs after exposure to ENMs. Based on the results described, it appears that simple cell cultures might be useful for prediction of adverse effects in vivo, whereas more complex cell culture models do not seem to increase the predictivity remarkably beyond the simple cell culture systems. At the same time, the results show that exposure of cells in submerged condition could be valuable in correlation analysis between in vitro and in vivo responses in animal studies. Studies of toxicological effects in cells exposed to the same insoluble or soluble ENMs in submerged condition and at ALI have shown inconsistent results (Table 4). In addition, these observations are predominantly based on experiments using a limited panel of ENMs in each study. In fact, it seems that only one study has investigated multiple ENMs and that indicated the same ranking of toxicological effect by exposure to TiO_2_ and CeO_2_ in submerged condition and ALI system (Loret et al., 2016). The studies comparing effects in cells exposed in submerged conditions with those exposed in the ALI system can be categorized into studies that show a strongest effect in ALI (i.e. higher response at the same dose or lower dose for the same level of response), the opposite or equivocal results. There is similar distribution of studies that have strongest effect in ALI (four studies; Loret et al., 2016; Hilton et al., 2019; Diabaté et al., 2021: Lovén et al., 2021), strongest effect in submerged condition (three studies; Herzog et al., 2014; Panas et al., 2014; Mills‐Goodlet et al., 2020) and equivocal outcome of the comparison between ALI and submerged condition (six studies; Lenz et al., 2009; Lenz et al., 2013; Stoehr et al., 2015; Cappellini et al., 2020; Medina‐Reyes et al., 2020; Di Ianni, Erdem, et al., 2021). This suggests that it is presently unclear whether the magnitude of effects with the ALI system is higher and whether such exposure systems are superior to submerged conditions. It is our impression that refinements (or improvements) have occurred for both ALI and submerged exposure systems over the period of from 2009 to 2021 (i.e., studies in Table 4). For instance, dispersion protocols are more standardized today than they were 10–15 years ago. The heterogeneity of results in Table 4 suggests that the optimal way forward to assess differences in responses in ALI and exposure condition would be to test the reproducibility of results by using the same ENMs (and same dispersion protocol), cell types, and biomarkers in different laboratories. On the same note, it would be desirable to test the reproducibility of correlations in vitro and in vivo responses in standardized conditions where different laboratories do these tests in parallel.

**TABLE 4:** Comparison of toxicological effects in cells models using exposure in submerged condition or air–liquid interphase

Author	Exposure model	Engineered nanomaterial	Cell type	Response
Lenz et al. (2009)	ALICE	ZnO	A549	Equivocal response (strongest response on *IL‐8* expression in submerged condition; strongest response on *HO1* expression by exposure at the ALI)
Lenz et al. (2013)	Not applicable	ZnO	A549	Equivocal response (strongest response on *IL‐8* expression in submerged condition; strongest response on *HO1* expression by exposure at the ALI)
Herzog et al. (2014)	ALICE	Ag	Co‐culture (A549, dendritic cells and macrophages)	*Lower* biological response by exposure at ALI than submerged condition
Panas et al. (2014)	Invitrocell	SiO_2_ (Aerosil200, sample with 50 nm)	A549	Biological effects observed at *higher* doses in ALI than in submerged condition
Stoehr et al. (2015)	ALICE	ZnO	A549 (with pIL8‐Luc vector)	Equivocal response (depending on measurement of *IL‐8* gene expression or protein levels)
Loret et al. (2016)	Invitrocell	TiO_2_ (NM‐105, NM‐101, NM‐110) and CeO_2_ (NM‐212)	Co‐culture (A549‐ and THP‐1a)	Biological effects observed at *lower* doses in ALI than in submerged condition
Hilton et al. (2019)	Invitrocell	MWCNT‐7	Co‐culture (A549, THP‐1a and MRC‐5)	Biological effects (proteomics) observed at *lower* doses in ALI than in submerged condition
Cappellini et al. (2020)	XposeALI	CeO_2_ (NM‐212)	Co‐culture (A549 and THP‐1a)	*Similar* level of toxicological effects in ALI and submerged exposure condition
Diabaté et al. (2021)	Invitrocell	CeO_2_ (three different samples), TiO_2_	A549	Biological effects (proteomics) observed at *lower* doses in ALI than in submerged condition
Medina‐Reyes et al. (2020)	Invitrocell	TiO_2_ (nanoparticles and nanofibers)	A549	*Similar* level of toxicological effects in ALI and submerged exposure condition
Mills‐Goodlet et al. (2020)	Invitrocell	SiO_2_ (and allergen co‐exposure)	Human alveolar epithelial lentivirus immortalized cells (hAELVi)	Biological effects (proinflammatory response) *not observed* in ALI at dose that caused effect in submerged condition
Di Ianni, Erdem, et al. (2021)	Invitrocell	MWCNT (NM‐400 and NM‐401)	Co‐culture (A549, THP‐1a, and WI‐38)	*Equal* biological response by exposure at ALI and submerged condition when comparing effective, deposited doses per surface area
Lovén et al. (2021)	NACIVT	ZnO	A549	Biological effects (proteomics) observed at *lower* doses in ALI than in submerged condition

In vitro models can also include co‐culture of multiple cell lines seeded in a 3D‐orientation to study the effects of ENMs (Klein et al., 2013), and similar models have been used to model profibrotic potential of MWCNT in vitro following repeated exposures (Barosova et al., 2020). To further increase complexity, microfluidic systems could be used to culture cells in organ‐on‐chip. In this system, epithelial cells and macrophages are cultured on a membrane exposed to air, and the micro channels include circulating neutrophils. This models could resemble neutrophil influx to the apical side of the chip following ENM deposition (Benam et al., 2016). The microfluidic system has also peristaltic pumps, which compress and stretches the epithelium hence mimicking the movement of the lung during respiration. This system could prove valuable to detect toxicological effects caused by shape of, for example, flake‐like ENMs (Di Ianni et al., 2020). In addition, advanced models could include sensitized models that recapitulate the pathophysiology of chronic obstructive pulmonary disease or asthma (Benam et al., 2016; Jimenez‐Valdes et al., 2020). Despite the promises of more complex models, there is still a lack of studies showing in vitro–in vivo correlations with more complex in vitro models and therefore more studies are necessary to draw conclusion on their predictivity.

The studies described in this review compare responses in vitro to the responses in vivo following exposure of animals by intratracheal instillation (12 studies), inhalation (3 studies), or oropharyngeal aspiration (5). Intratracheal instillation is broadly used as surrogate for inhalation and has the advantage of delivering the suspended ENMs as a bolus, with known amount of delivered ENM which, on the other hand, would need to be quantified or computationally modeled in the case of exposure via inhalation. Nevertheless, compared with inhalation, instillation is not a physiologically relevant exposure, has higher dose rate, and may cause a different distribution of deposited doses. Further, instillation may cause stronger inflammatory responses compared with inhalation, as it was shown for carbon black at similar deposited doses (Jackson et al., 2012). In oropharyngeal aspiration, the substance is placed in the pharynx, which is more proximal than the trachea in the respiratory tract. The deposition pattern of pharyngeal aspiration is more dispersed than that of intratracheal instillation, making it more similar to inhalation (Kinaret et al., 2017). Using the same surface dose, previous studies showed higher inflammatory and tumor response by instillation than inhalation (Møller et al., 2013; Osier & Oberdörster, 1997). The concern about delivered doses in animal studies leads to a general concern about the assessment of uncertainty in studies on the correlation of responses in vitro and in vivo. For in vitro studies, there might be uncertainty about the delivered dose in submerged exposure condition (i.e., the dose depends on the sedimentation rate), whereas the deposited dose is determined as a part of the experiment in the ALI system. On the contrary, there might be uncertainty about the delivered dose in inhalation studies (i.e., deposition depends on the physico‐chemical characteristics of ENMs), whereas the delivered dose is certain in studies on intratracheal instillation. Thus, depending on the comparison, there can be uncertain about the dose of the predictor (i.e., submerged condition) and dependent (i.e., inhalation) variable in statistical analysis. It suggests that the best studies—in terms of alleviating nondifferential exposure misclassification—would be exposure at the ALI versus the same responses in animals after intratracheal instillation. As nondifferential exposure misclassification blunts associations between predictor and outcome in statistical analysis, it can be speculated that studies using submerged condition and inhalation exposure may give rise to lower correlation coefficients or concordances than studies using exposure by ALI and intratracheal instillation.

The determination of effective doses, as well as use of surface area‐metric, might improve the assessment of hazard rankings in vitro. Very importantly though, there are too few studies to firmly draw conclusions on whether or not complex cell culture models are superior in predicting in vivo responses. However, based on what is discussed here, there are different approaches that are promising to advance the in vitro–in vivo comparisons of ENMs in the nanotoxicology field and beyond. In general, the ALI systems are still in stages of being validated, although intra‐ and interlaboratory reproducibility of the CULTEX system has been tested on relatively large panel of compounds, including ENMs (Steinritz et al., 2013; Tsoutsoulopoulos et al., 2019). It should also be noted that the studies so far have assessed biomarkers as proxy‐measures of pulmonary diseases. The association between toxicity of ENMs in cell cultures and hard endpoints such as pulmonary fibrosis and neoplastic diseases is unresolved. For instance, evidence suggests that certain ENMs, such as TiO_2_ and carbon black, are not carcinogenic in mice following inhalation exposure, whereas rats appear to be a sensitive species. The extrapolation from in vitro and animal models to human hazard assessment may be aided by investigating benchmark particles for which epidemiological and long‐term animal studies are available. These include quartz, diesel exhaust particles, and asbestos (IARC, 2012, 2013).

A lot has been discussed about the realistic dose ranges in vitro. For example, 160 μg/ml or 45.7 μg/cm^2^ in Di Ianni, Erdem, et al., 2021 are much larger compared with instilled doses in vivo (e.g., up to 162 μg or 2 μg/cm^2^ of the alveolar surface area [mouse alveolar surface area 82 cm^2^; Knust & Ochs, 2009]). In a recent report, Ma‐Hock et al. (2021) proposed a method to use in vivo organ burden to define the doses to be tested in vitro. Specifically, the lung burden at the LOEC would be used for defining the effective dose to which the cells should be exposed to detect a response. For the case of CeO_2_, for example, the in vitro effective concentration range should span around 0.003 μg/cm^2^ based on extrapolation of the doses used in animals (Ma‐Hock et al., 2021). In perspective, Loret et al. (2018) found the BMD of cytokines induction (IL‐6, IL‐8, IL‐1β, and TNF‐α) in vitro to be 100–200 times larger than the BMD of the same cytokines in vivo. If the BMDs in vivo shall be applied to in vitro studies, there could be potentially no effect also for particles that are known to be highly potent. Similarly, Teeguarden et al. (2014) showed that higher doses of Fe_3_O_4_ were needed to induce inflammatory response in macrophages in vitro (~1.2–4 μg/cm^2^ cell surface area) than when administered in vivo (0.009–0.4 μg/cm^2^ alveolar surface area). Jagiello et al. (2021) determined the BMD to activate inflammatory reactions involved in the development of lung fibrosis (Adverse outcome pathway nr. 173) by exposure to MWCNT, for example, the BMD of a specific type of MWCNT called NM‐401 was 0.73 μg in mice, corresponding to 0.0088 μg/cm^2^ of alveolar surface area. This value is still way far from the doses of NM‐401 that induced significant effects in submerged conditions (effective doses) and in the ALI (Di Ianni, Erdem, et al., 2021). Accordingly, the dose range tested, minimum 5.25 and 4.45 μg/cm^2^ were necessary to induce a significant effect in A549 and THP‐1a cells, respectively, and 9 μg/cm^2^ to the co‐culture at the ALI (Di Ianni, Erdem, et al., 2021). It is important to note that the lung complexity in vivo is much higher compared with the mono‐ and co‐culture models creating a large differences in terms of doses required to induce effects. Moreover, the meaningfulness of comparing “realistic exposures” in cell cultures to exposures in animals or humans has been questioned as primary cells undergo a “culture shock” when they are cultured outside the body and those that survive are adapted to ex vivo conditions. In addition, something as simple as the doubling time of the same cell line, obtained in different laboratories, has been shown to differ substantially (Møller et al., 2021). The inclusion of benchmark particles with known toxicity in vivo and in humans may provide scaling between in vitro and in vivo responses.

The mechanism of action and the target cells following ENMs exposure can be pivotal for identifying the right models and select biomarkers of toxicity. Adverse outcome pathways (AOP) are OECD frameworks describing the mechanism of action (oxidative stress, inflammation, genotoxicity, etc.) of a compound into a series of key events (KE) leading to an adverse outcome (AO), via one or multiple KE, interconnected by causative relationships (Halappanavar et al., 2020). AOP are developed based on analyses of tissue histological changes as well as omics. Further, AOP can also guide the selection of in vitro models to be used in the in vitro assessment. Namely, single cell RNA sequencing (sc‐RNA‐seq) might be a valuable tool in the development of in vitro models, by identifying the animal lung cell subpopulations that are most reactive, with gene upregulations, following particle deposition in the respiratory regions. These cells and the specific genes could be used in the quantification of ENM‐caused hazard in vitro, with in vitro results correlated to the responses in vivo. By knowing the relationship between different KEs (e.g., proinflammatory upregulation and recruitment of neutrophil influx), AOP can guide the selection of in vitro models and endpoints for the assessment of KE, including biomarkers such as neutrophil chemoattractants (IL‐8) in vitro and neutrophil influx in vivo (Di Ianni, Erdem, et al., 2021; Di Ianni, Møller, et al., 2021). In line with this, it has been postulated that battery tests of KE leading to AO could ultimately be included in tiered testing strategies that bypass the use of animals for characterization of ENM hazards (Halappanavar et al., 2020, 2021; Nymark et al., 2021). The mechanism‐based hazard assessment aided by AOPs could be pivotal in New Approach Methodologies (NAMs), which represent, according to the European Chemical Agency, “*a broad context that includes in silico approaches*, *in chemico*, *and in vitro assays*, *as well as the inclusion of information from the exposure of chemicals in the context of risk assessment*.” Divided in different categories, NAMs are meant to reduce or replace the use of animals in high dose toxicity studies (Nymark et al., 2020). These categories are high‐throughput screenings, grouping and read across, exposure assessment, and modeling. Quantitative structure–activity relationships, AOPs, dose–response extrapolations, and benchmark dose identification (including EPA method and RIVM PROAST (Slob & Setzer, 2013).

The studies included in this review focused on toxicity of well‐characterized ENMs. However, the methods discussed here might be applied to complex mixtures of nanoparticles, such as those in particulate matter from urban environment or occupational settings. Breznan and colleagues explored the relationship between particle effects in vitro and in vivo following exposure to a group of urban particles, minerals and ENMs. By correlating the individual in vitro and in vivo particle potency estimates, as well as the in vitro and in vivo combined particle potency estimates, the authors observed that the correlations of combined inflammatory potential in vitro and vivo, and the integrated potency estimates in cells versus mice approached the threshold for significance *α* = 0.05 (*r*_*s*_ = 0.8, *p* = 0.052) (Breznan et al., 2016).

The present review has described associations between responses in cells and animal lungs, which have been highlighted in the selected publications. In certain instances, the authors have assessed multiple biomarkers in cells, but only focused on the positive associations. Pal et al. (2015) is one such example where strength of the correlation between cytotoxicity (in vitro) and pulmonary inflammation (in vivo) was highlighted, but the correlations between proinflammatory responses in cells and animals were not mentioned. Based on the slopes reported in the article, there was the same ranking of PMN influx in animals and in vitro cell viability (Printex 90 > Ni InCo > nanoAg > CeO_2_ > TiO_2_), whereas IL‐8 release had a different ranking (Printex 90 > nanoAg > Ni InCo > CeO_2_ > TiO_2_). Another example is Cui et al. (2019) that focuses on responses in metabolites in vitro and in vivo; however, the publication also contain data that indicate a lack of consistency in proinflammatory responses in cells and animals. A future approach could be to assess hypothesis‐driven associations, where the use of relevant biomarkers is justified before the experimental work is performed. The two examples suggest a risk of reporting bias in the present dataset. In fact, reporting, publication and citation bias need to be carefully assessed in systematic reviews of the association between in vitro and in vivo responses after exposure to ENMs. Null effect or inconsistent findings might be detected in studies with multiple objectives. For instance, reporting bias has been observed in a systematic review of genotoxic effects by TiO_2_ as a somewhat higher proportion of positive findings in studies on only TiO_2_ as compared with studies with multiple ENMs (Møller et al., 2017). Publication bias can be difficult to detect, but one way is to look for asymmetry in reported effect sizes versus the study size or precision (e.g., Funnel plots) or assessing the relationship between reports in peer‐review journals and other kinds of disseminations (e.g., conference proceedings or project delivery reports). As an example, publication bias has been speculated to occur in studies on genotoxicity of MWCNTs as null effect finding in EU projects do not appear to published at the same rate as effects on genotoxicity (Møller et al., 2021). Reporting bias may occur if authors selectively cite other studies that have reported a positive association between in vitro and in vivo findings. A way to assess reporting bias is to compare citations of articles with opposite findings as for instance has been done in systematic review of DNA damage results by the comet assay (Møller et al., 2010, 2020). Publication and citation biases are flaws that may skew the interpretation of experimental evidence in a certain direction. Thus, it is important to proceed in systematic reviews with predefined hypotheses. Meta‐analyses may only be included if there are sufficient number of studies with reasonably similar study design and biomarkers. Publications in the current database are too heterogeneous to be used in meta‐analyses and we have used a somewhat explorative search strategy. Especially, it seems prudent to standardize the association between predictors and outcomes. At least three different approaches have been used in the 18 studies that we have systematically evaluated, including binary and continuous outcomes of the biomarkers (e.g., effect size of cytokine release), and doses at fixed effect of biomarkers.

In summary, studies using simple in vitro models with cells exposed to ENMs in submerged condition have been shown to predict the extent of the toxic effects in vivo. There are relatively few studies that have assessed predictivity of the complex models, including ALI, and they have not indicated associations stronger than those observed with simple monoculture systems.

## CONFLICT OF INTEREST

The authors declare no conflict of interest.

## AUTHOR CONTRIBUTIONS

**Emilio Di Ianni:** Conceptualization (equal); data curation (lead); investigation (lead); methodology (lead); writing – original draft (lead); writing – review and editing (lead). **Nicklas Raun Jacobsen:** Conceptualization (equal); funding acquisition (equal); methodology (equal); supervision (equal); writing – review and editing (equal). **Ulla Vogel:** Conceptualization (equal); funding acquisition (equal); supervision (equal); writing – review and editing (equal). **Peter Møller:** Conceptualization (equal); data curation (equal); formal analysis (equal); investigation (equal); methodology (equal); supervision (lead); writing – original draft (equal); writing – review and editing (equal).

## RELATED WIRES ARTICLES

Nanoparticle dosage‐a nontrivial task of utmost importance for quantitative nanosafety research

High throughput toxicity screening and intracellular detection of nanomaterials

Toward toxicity testing of nanomaterials in the 21st century: A paradigm for moving forward

## Data Availability

Data available on request from the authors.
